# Molecular Orbital Insights of Transition Metal-Stabilized Carbocations

**DOI:** 10.3389/fchem.2019.00365

**Published:** 2019-06-04

**Authors:** Hannah Goodman, Liangyong Mei, Thomas L. Gianetti

**Affiliations:** Department of Chemistry and Biochemistry, University of Arizona, Tucson, AZ, United States

**Keywords:** metal complexes, carbocation, bonding model, metal-carbocation interaction, molecular orbital interactions

## Abstract

Transition metal-stabilized carbocations are characterized by synthetically valuable interactions, yet, to date there are no comprehensive reports of the many bonding modes that can exist between a metal and carbocation. This review summarizes developments in these complexes to provide a clear picture of their properties and reactivities. In order to strategically exploit them, we propose this summary of the different bonding modes for transition metal-carbocation complexes. These models will help chemists understand the orbital interactions involved in these compounds so that they can approach their synthetic goals most effectively. Multiple transition metals and carbocations will be discussed.

## Introduction

**Figure 1:**
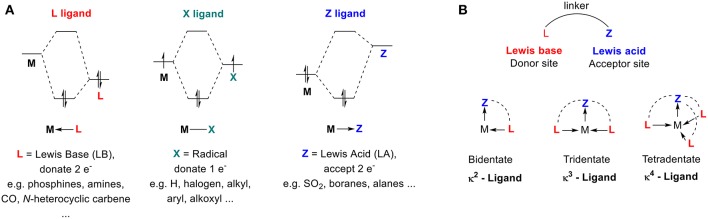
**(A)** Simplified orbital diagrams associated with M—L, M—X, and M—Z interaction. **(B)** Schematic representation of ambiphilic ligands.

The structures, properties, and reactivities of organometallic complexes depend mostly on their ligand environment. Ligands are used to improve catalyst efficiency and accelerate the discovery of new reactivity modes. We will use M. Green's seminal model for Covalent Bond Classification (CBC method) (Green, 1995; Parkin, 2007) to define 2-center metal-ligand interactions of organometallic compounds as ML_l_X_x_Z_z_, where the ligand atoms are classified as L, X, and Z ligands (Figure 1A). L-type ligands are Lewis bases that donate two electrons to form a dative L → M bond (e.g., PR_3_, NR_3_, OR). X-type ligands donate one electron, requiring oxidation of the metal center to form classical covalent M–X bonds (e.g., H^−^, RO^−^, Cl^−^). Z-type ligands are Lewis acids that accept a pair of electrons from the metal to form a dative M ← Z bond (e.g., SO_2_, BR_3_). Since transition metals (TMs) are typically defined as electron-deficient species, the majority of ligands that have been developed are electron-rich Lewis-basic moieties (L- and X-types) that aim to complete the valence shell of the transition metal (also known as the 18 electron rule). However, transition metals also exhibit Lewis-basic character from metal-to-ligand back-donation from partial filling of their upper valence d shell. Organometallic chemists have recognized how this basicity can be used to promote interactions between a TM and a Lewis acid moiety, where the acid acts as a σ-acceptor (L-type) and not a σ-donor (Z-type) ligand.

The first example of Lewis acid–metal coordination was reported in 1970 (Shriver, 1970), yet the incidence of M ← Z complexes remained scarce for several decades. The scope of Lewis acids acting as σ-acceptor ligands has been significantly extended over the last two decades with the development of ambiphilic ligands—ligands that contain both electron donor (L-type) and acceptor (Z-type) groups (Amgoune and Bourissou, 2011; Braunschweig and Dewhurst, 2011; Owen, 2012, 2016; Bouhadir and Bourissou, 2016; Jones and Gabbaï, 2016). Transition metals exhibit similar ambiphilic character since they contain both filled and empty d-orbitals. When ambiphilic ligands are used, the metal center coordinates to the L-type moiety of the ligand and the degree of coordination to the Lewis acid moiety is increased (Figure 1B). The Z-type ligand can stabilize a vacant orbital of the transition metal center while drawing electron density from the filled d-orbitals because of its σ-accepting properties. This interaction determines the electronic properties and reactivities of the metal (You et al., 2018). The synthesis, coordination chemistry, and reactivity of ambiphilic ligands and their metal complexes have been extensively studied and well-summarized in several recent reviews (Amgoune and Bourissou, 2011; Braunschweig and Dewhurst, 2011; Owen, 2012, 2016; Bouhadir and Bourissou, 2016; Jones and Gabbaï, 2016). Surprisingly, none of these reports have mentioned carbocations as Lewis acid Z-type ligands, and they neglect to examine the interactions between transition metals and persistent carbocations.

In organic chemistry, carbocations are ubiquitous. The field of carbocation chemistry has rapidly developed since its conception in 1901 when Norris discovered the first stable carbocations, triphenylmethyl ions (PH_3_C^+^) (Norris, 1901; Norris and Sanders, 1901) Carbocations have been identified as key intermediates in many organic reactions, including electrophilic aromatic substitutions, unimolecular nucleophilic substitutions, addition-eliminations, and many rearrangements (Olah, 2004). The significance of carbocations as reactive intermediates in acid-mediated reactions was also highlighted when Professor Olah was awarded the Nobel Prize in Chemistry to in 1994 for his contributions to carbocation chemistry (Olah, 1995). The diverse applications of carbocations in organic chemistry is outside of the scope of this review, but interested readers are encouraged to consult the many reviews published on their synthesis and applications.

**Figure 2:**
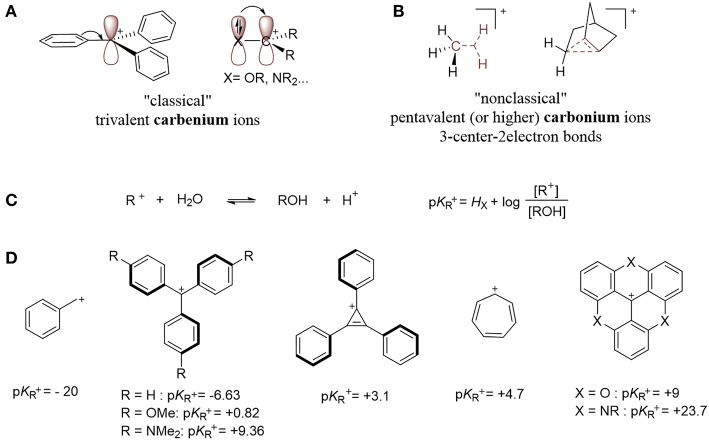
**(A)** Examples of carbenium ions. **(B)** Examples of carbonium species. **(C)** Determination of carbocation stability: p*K*_R+_. **(D)** Examples of carbocations and their p*K*_R_^+^ values (Breslow and Chang, 1961; Ritchie, 1986; Amyes et al., 1992; Sørensen et al., 2014).

This review focuses on organometallic complexes that contain a carbocation (Z-type ligand), within their first coordination sphere. In 1972, Olah proposed carbenium and carbonium ions as two distinct types of carbocations that are differentiated by their structures (Olah, 1972). The “classical” trivalent carbenium ion contains an *sp*^2^-hybridized electron-deficient carbon atom (Figure 2A), while the “non-classical” carbonium ion is defined as a penta- (or higher) coordinate carbon that involves a 3-center-2-electron bond (Figure 2B; Winstein and Trifan, 1952; Olah, 1972). This review focuses on classical carbenium ions, which we will refer to as carbocations.

Carbocations can be transient, extremely reactive species, or they can be long-living, isolable, and storable. They gain stability from filled p orbitals or π-systems α to a *sp*^2^ hybridized carbon, which creates resonance and delocalizes the positive charge over multiple atoms (Figure 2A). Their thermodynamic stability can be described in term of their p*K*_R_^+^ value, which is defined by the equilibrium between the cationic species and its corresponding carbinol (Figure 2C; Deno et al., 1955) A larger p*K*_R_^+^ value correlates to a more stable carbenium or, specifically, one that is resistant to nucleophilic attack by water. Examples of persistent carbenium ions and their p*K*_R_^+^ values are shown below (Figure 2D; Breslow and Chang, 1961; Ritchie, 1986; Amyes et al., 1992; Sørensen et al., 2014).

## General Bonding Considerations

**Figure 3:**
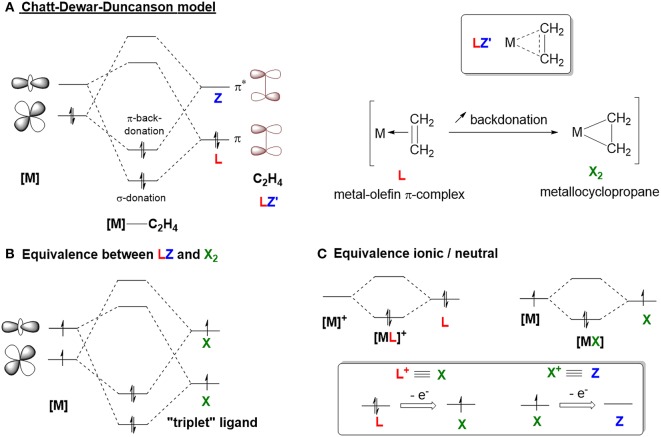
**(A)** Chatt-Deward-Duncanson model. **(B,C)** Covalent bond classification equivalency.

The bonding mode for most of the Z-type ligands described in the literature is unambiguous (Amgoune and Bourissou, 2011; Owen, 2012). In these models, there is a localized empty p orbital that acts as a σ-acceptor for a filled metal-based d orbital on either a group 13 or a hypervalent, heavy group 14 element (Figure 1A). However, this bonding model becomes more complicated when considering carbocation-containing species since the empty *sp*^2^ hybridized orbital is often involved in an extended π-system that can act as an L- or X-type ligand. In order to discuss the bonding model of carbocations in depth, it is important to remind the reader about an important concept—donation and backdonation. This concept is represented by the Dewar-Chatt-Duncanson model (Figure 3A) with regard to metal-olefin interactions (Nelson et al., 1969; Mingos, 2001; Frenking, 2002). The filled π-orbital of the olefin donates electron density to the metal center via an interaction with an empty metal-based d orbital. This donation (L-type) is supplemented by backdonation from a filled metal-based d orbital into the empty π^*^-orbital (Z-type). Figure 3A shows how the complex can be described either as a metal-olefin adduct from modest backbonding (resulting in an L-type ligand), or as a metallacyclopropane derivative due to extensive backbonding (where the olefin serves as an LZ ligand, otherwise known as an X_2_ ligand). The equivalence between an LZ and X_2_ system comes from the fact that both types require the involvement of two metal orbitals (Figure 3B; Parkin, 2007). Similar to the relationship between Fischer carbenes and Schrock alkylidenes (Fischer, 1976; Schrock, 2001, 2005; Shrock, 2002), the “triplet” state of the ligand becomes accessible only if the empty orbital is sufficiently low in energy. However, both bonding descriptions represent extreme cases of this model and most reported olefin complexes are more accurately described as a hybrid, with varying degrees of backdonation (Figure 3A, LZ′). The extent of backdonation strongly depends on the nature of the metal center. For example, a metal with a pair of electrons residing in a high energy orbital will favor strong backbonding interactions because of energy matching. Since it is impossible to predict which model is preferred without further spectroscopic or structural analysis, Parkin introduced the LZ′ designation, where Z′ refers to an unspecified degree of backbonding (Parkin, 2007). Examples of these types of ligands are C_2_H_4_ and CO. In order to describe the bonding mode for carbocation-containing ligands, we will employ this Z′ classification.

Another important concept is the equivalent neutral class (Figure 3C; Parkin, 2007) the classification of the ligand changes with the presence of any charge or delocalization of charge. A donor ligand with x-electrons bound to a cationic metal center is equivalent to a donor ligand (x-1)-electrons bound to a neutral metal center (i.e., [ML]^+^ ≡ [MX], so X^+^ ≡ Z). Due to the cationic nature of the ligand of interest, this notation will be used to differentiate between partial backdonation (Z') and strong backdonation/charge delocalization (X^+^).

**Figure 4:**
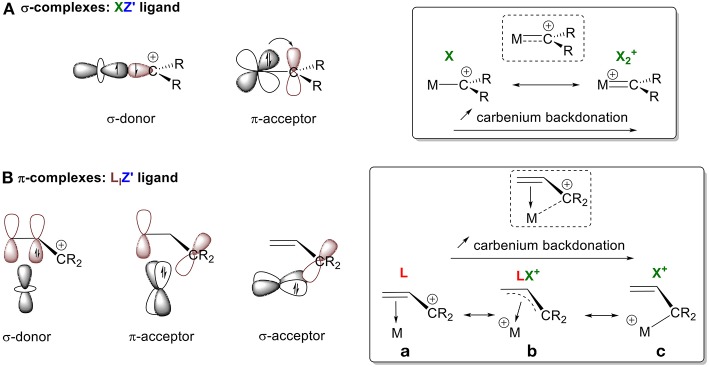
Orbital interactions between a carbenium ion and a metal center in **(A)** σ-complexes, **(B)** π-complexes.

Carbocation species can form either σ- or π-complexes depending on whether they are carbenylium (R_2_C^+^) or carbenium (R_3_C^+^) moieties. Carbenylium R_2_C^+^ ions possess a half-filled *sp*^2^ orbital that can participate in σ-bonding with a metal center. They also have an empty p orbital, which is available for backdonation (Figure 4A). Using the CBC method, R_2_C^+^ can be described as an XZ′ ligand. These molecules are analogous to LZ′ carbenes R_2_C: (vide supra, L^+^ → X). These carbenes can be classified in two ways, depending on the extent of π-backbonding from the metal center: either as a “Fischer carbene,” which is an L-type ligand due to weak backdonation, or as a “Schrock alkylidene,” which is an X_2_ ligand because of strong backdonation (Parkin, 2007). A similar description for the interaction between a carbenylium R_2_C^+^ and a metal center can be used that also depends on the extent of π-backdonation. Stabilization of the empty p orbital by an alkyl substituent will result in no backdonation; in this case, R_2_C^+^ is a pure X ligand (i.e., X = L^+^ for comparison with a Fischer carbene). On the other hand, no stabilization results in extensive backbonding so that R_2_C^+^ is classified as and XZ ligand (i.e., XZ = X_2_+ for comparison with a Schrock alkylidene). Types of interactions between these extreme configurations can also be observed (XZ′ function, Figure 3A), as illustrated by our olefin model.

Carbenium R_3_C^+^ moieties only possess an empty p orbital, resulting in the formation of π-complexes (Figure 4B). For consistency in our discussion, we describe their bonding modes, differentiated by the extent of interaction between the metal orbitals and carbon's empty 2p orbitals. These modes of interaction range from: (1) no C^+^ interaction, (2) weak interactions, and (3) full hybridization of the carbon. The persistent carbenium ions that will be discussed are stabilized by resonance with an adjacent π-system. Therefore, the ligand is considered ambiphilic, since it acts both as a σ-donor through its electron rich π-system (L-type ligand) and as a π and/or σ-acceptor through its empty π^*^-system and p orbital (Z-type ligand). Again, the bonding mode depends on the electron accepting ability of the ligand framework. In the absence of σ-backdonation from the metal, the carbenium species acts as an L-type ligand and does not interact with the metal center. In the absence of σ-backdonation from the carbenium, interactions between the filled π-system and the metal center will be the main orbital interactions, leading to a η^2^ coordination mode with an L-type ligand (Figure 4B,a). As the interaction between the metal and the empty p orbital of the carbenium increases, the allyl cation acts as an LX^+^ ligand through π- and/or σ-backdonation, resulting in η^3^ bonding (Figure 4B,b). In the presence of extended σ-backbonding into the p orbital of R_3_C^+^, the M-C^+^ interaction will govern the bonding mode leading to full hybridization of the carbon and a η^1^ coordination mode (Figure 4B,c). This simplistic model is used to introduce the notion of Z′ and relative backdonation into carbenium π-complexes. A more relevant bonding view of each example system will be further discussed in their corresponding sections. This will allow us to address how the Z′ character of a carbenium ligand is affected by the nature of the metal, its d electron count, its coordination environment, and the π-systems that stabilize the C^+^ atom.

## σ-Complexes

As stated in the initial bonding discussion, σ-bonded carbenium complexes are in equilibrium with a carbene-bonded cationic metal. Carbenylium cations and Fisher carbenes have similar bonding modes and are considered π-acceptors. By analogy, cyclopropenylium ions are XZ'-type ligands and Fisher carbenes are LZ'-type. However, carbocations donate one electron as σ-donors while carbenes donate two electrons as σ-donors. Carbene complexes are prevalent, well-discussed species in literature so we will not focus on presenting the extensive progress in the field of carbene chemistry. Interested readers are encouraged to review articles on this subject (Ofele et al., 2009; Melaimi et al., 2010; Martin et al., 2011; Moerdyk and Bielawski, 2013). Instead, we will use selected examples to generalize bonding interactions between carbenium ions and metal centers in a σ-manner, and highlight how this bonding model is affected by the nature of the R groups on the carbenium and the metal and its ligands (L_n_).

### Cyclopropenylium Cations

Cyclopropenylium cation (C_3_H_3_^+^) is the smallest member of the Hückel aromatic system (Breslow, 1957; Breslow and Chang, 1961). It exhibits considerable thermodynamic stability from aromaticity and resonance with 2 π-electrons delocalized over three conjugated 2p orbitals. The symmetry of its π-system imparts enormous stability relative to typical carbocations. Its protonated analog, cyclopropene, is a strained three-membered ring that is hugely thermodynamically unstable. The stability of the cation relative to the instability of the neutral species has elicited great interest in chemists and inspired synthetic and theoretical studies for decades. Cyclopropenylium cations were first synthesized by Breslow (1957) when he synthesized triphenylcyclopropenylium cation (Breslow, 1957). This was the first experimental verification of aromaticity in non-benzenoid molecules and it offered an important lesson: the energetic debt from ring strain can be compensated by aromatic stability (Breslow and Chang, 1961). Although the cation has been widely investigated since its discovery, the number of metal-bound cyclopropenylium complexes is not as abundant. A thorough review of this topic was presented by Komatsu and Kitagawa (2003).

The first isolated σ-bound cyclopropenylium-metal complexes were reported in 1978 in consecutive articles by Gompper and Bartmann (1978) and by Weiss and Priesner (1978) following two different approaches.

**Figure 5:**
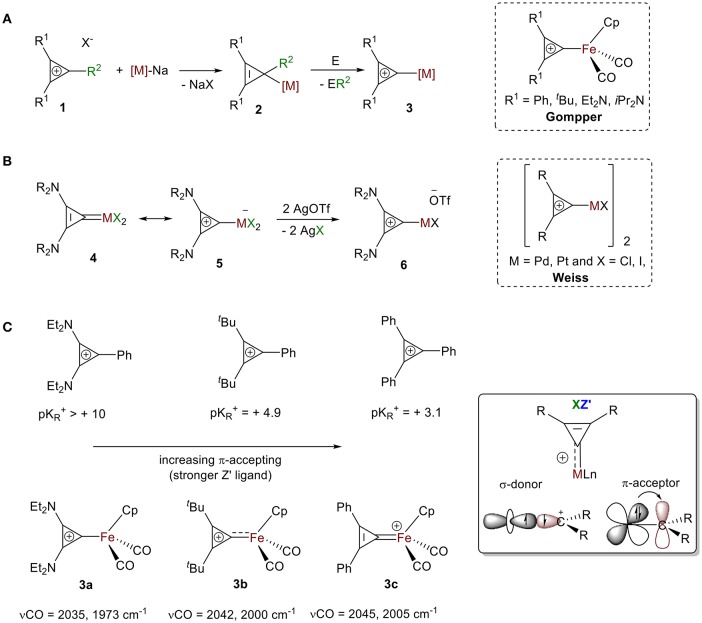
General synthesis for cyclopropenylium complexes: **(A)** Approach 1, **(B)** Approach 2. **(C)** Influence of the ring substituent on the extent of back donation.

**Approach 1:** Bartmann reported the synthesis of dicarbonyl(η-cyclopentadienyl)(σ-2,3-diphenylcyclopropenyl)iron salts **2** from the nucleophilic attack of a coordinatively unsaturated metallate, sodium dicarbonyl(η-cyclopentadienyl)ferrate salt, to various cylcopropenium ions **1** (Figure 5A). While the formation of NaX (X = cyclopropenium counter ions) is a strong driving force for this reaction, the neutral compound **2** is highly strained and reactive from loss of aromaticity. Stability from aromaticity was easily restored by abstraction of the R_2_ group (e.g., Ph, He, H, Cl) with appropriate abstracting agent E (e.g., HCl, I_2_, [(Ph)_3_C^+^][BF_4_^-^]), resulting in the σ-bound cyclopropenylium ion complexes **3** (Gompper and Bartmann, 1978).
**Approach 2:** Weiss and Priesner proposed that the neutral cyclopropenylidene complex **4**, first reported by Öfele (1970), is in resonance with the Zwitterionic form **5**, where a metallate anion is bound to a cyclopropenylium moiety (Figure 5B; Weiss and Priesner, 1978). With the aid of a strong Lewis acid, bound anionic ligand X^−^ can be exchanged with a non-coordinating anion (e.g., ^−^OTf), leading to the formation of transition metal-substituted cyclopropenylium system **6**. A variety of cyclopropenylidene complexes have since been reported. Their synthesis typically follows one of three routes: (1) oxidative addition of dihalocyclypropenes followed by halide abstraction (Öfele, 1970; Konishi et al., 1978; Weiss and Priesner, 1978; Yoshida, 1982; Miki et al., 1988), (2) reaction of the cyclopropenyl salts with lithium adducts (Rees and von Angerer, 1972; Gompper and Bartmann, 1978; Konishi et al., 1978; Yoshida, 1982; Miki et al., 1988; Tamm et al., 1995; Schumann et al., 1997), or (3) cyclopropenylidene transfer (Yoshida, 1982; Gade et al., 2000; Kozma et al., 2013). These carbene species have been extensively discussed and were thoroughly reviewed by Herrmann in 2009 (Ofele et al., 2009). The bonding mode of cyclopropenylidene complexes is unambiguous and well-established by carbene chemistry and, therefore, will not be presented in this review.

As discussed, the extent of π-backdonation depends on the energies of the overlapping orbitals. Substituents on the ring alter the XZ bonding mode for this ligand because of the impact they have on the extent of electron donation and π-backdonation to the ring. In their initial report, Gompper and Bartmann synthesized complexes of substituted cyclopropenium ions with [Fe(Cp)(CO)_2_] (Gompper and Bartmann, 1978). They measured strength of the interaction between the iron atom and the carbon atom of the C_3_ ring with IR spectroscopy and found the stretching frequencies of the CO moiety bound to iron in **3a-c** showed a clear blueshift of the ν_(CO)_ between R = Ph, R = ^*t*^Bu, to R = NEt_2_ (Figure 5C), which is consistent with a decrease in π-backdonation from the metal into the CO ligand. This decreased electron density on the metal center is a result of the increased π-accepting ability of cyclopropenylium ligand, which correlates to a lower pK_R_^+^ value (Figure 5C). These trends support the XZ-type bonding model discussed in the first part of this review (vide supra, Figure 4A) and is analogous to the cyclopropenylidene complexes summarized in Herrmann's review: complexes bearing cyclopropenylidene ligands with two amino substituents showed the most σ-donor/least π-acceptor ability, and those containing two phenyl groups show the least σ-donor/most π-acceptor ability (Ofele et al., 2009).

This also supports our claim that that CR^2^^+^ acts as an XZ′ ligand with different degrees of Z-type interactions. More precisely, complexes with C(C(NR_2_)_2_)_2_^+^ ligands are best described as X-type ligands with little or no π-backdonation, while complexes containing ^+^C(CPh_2_)_2_ ligands are more appropriately represented as X_2_^+^ ligands because of their large Z-type interaction.

### Arylenium Cations

**Figure 6:**
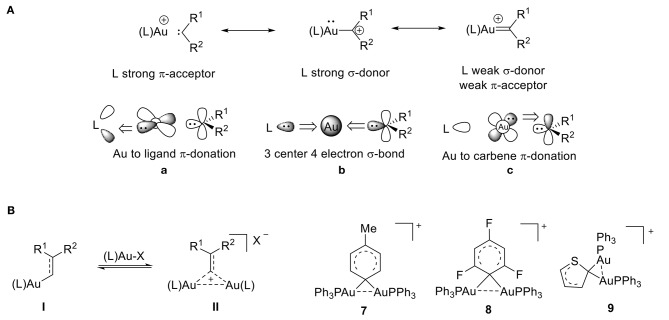
**(A)** Bonding model for gold-carbene resonance structures. **(B)** *Gem*-diaurated carbocation species.

Over the past decade, cationic gold(I) complexes have become some of the most efficient and versatile catalysts for the functionalization of C-C bonds (Echavarren and Nevado, 2004; Olah, 2004; Fürstner and Davies, 2007; Hashmi and Rudolph, 2008; Li et al., 2008; Jia and Bandini, 2015; Harris and Widenhoefer, 2016; Hopkinson et al., 2016; Zi and Toste, 2016; Shahzad et al., 2017). These complexes are typically formed from gold carbenes or from α-metallocarbenium ions. They contain a gold atom that is bound to a formally divalent carbon atom and are applied in a variety of gold-catalyzed transformations. The electronic structure of these cationic complexes is dependent on the extent of π-backbonding from the gold atom to the C_1_ carbon atom (Figure 6A). The bonding mode of gold carbene/carbenium complexes has been extensively discussed, and much of gold carbene complexes' behavior can be understood by applying the bonding model developed by Toste and Goddard (Benitez et al., 2009). According to this model, the L–Au–C bonding network is comprised of three sets of orbital interactions: (1) a three-center, four-electron σ-hyperbond that donates electron density from filled *sp*^x^ orbitals on the carbene's carbon atom to gold's empty 6s orbital (Figure 6A,b), (2) two orthogonal π-bonds that donate electron density from the metal's filled 5d orbitals to the ligand's π-acceptor orbitals (Figure 6A,a), and (3) the carbene's π-acceptor orbitals (Figure 6A,c). It follows that greater σ-donation from the ligand (L) results in a weaker σ component of the Au–C_1_ bond and greater π acidity of the ligand results in weaker Au-C_1_ backdonation.

Cationic gold(I) complexes have been extensively studied, so this review will focus only on what we consider to be one of the most intriguing species present during gold catalysis: the *gem*-diaurated carbocation species (a carbocation that is stabilized by two gold atoms through Au-Au contacts) (Hashmi, 2014). The abundance of cationic *gem*-diaurated species discovered in gold-mediated catalysis (Harris and Widenhoefer, 2016) helped researchers conclude that an equilibrium exists between the vinyl gold(I) species **I** and the *gem*-diaurated species **II** (Figure 6B; Roithová et al., 2012; Harris and Widenhoefer, 2016). Further, the substituents R^1^ and R^2^ influence this equilibrium. A balance of stability and reactivity is required to observe this diaurated species **II**; otherwise, **II** can form a catalyst-poisoning thermodynamic sink, rendering the equilibrium irreversible (Roithová et al., 2012). In order for efficient catalysis to take place, the equilibrium must be reversible, and the *gem*-diaurated species **II** is actually a less reactive off-cycle species than the corresponding vinyl gold(I) species **I** (Brown et al., 2012; Zhdanko and Maier, 2013). This can be rationalized by the fact that the *gem*-diaurated species **II** is stabilized through Au-Au interactions, which makes it less reactive than the unstabilized vinyl gold(I) species **I**.

The tolyl complex **7** represents the first 1,1-diaurated carbocation derived from benzene (Nesmeyanov et al., 1974) and the cation of perchlorate salt **8** provided the first crystallographic evidence for 1,1-diauration (Figure 6B; Rafael et al., 1988). According to its X-ray data, complex 8 shows an Au-C-Au bond angle of 79.3°, Au-C bond lengths of 2.16 Å, and a relatively short Au^…^Au distance of 2.76 Å. The two [AuPPh_3_] units in the diaurated thienyl complex **9** have identical phosphorus environments based on the ^31^P NMR, which shows only one peak. The structure includes a short Au^…^Au distance of 2.81 Å and a small Au-C-Au angle of 82.5° (Porter et al., 2003), which shows a strong aurophilic interaction (Schmidbaur, 1990; Mond et al., 1995; Stephen et al., 2012), that is consistent with other reports of diaurated compounds (Osawa et al., 2008; Seidel et al., 2010). The literature range of Au-Au distances for gem-diaurated compounds is 2.72 to 2.85 Å.

## π-Complexes

### Cyclic Carbocations

#### Cyclopropenium Cations

The first π-complexes with cyclopropenium cation were synthesized by Hayter (1968). His brief report included the synthesis of only one cyclopropenium-ligated complex, [(π-C_5_H_5_)Mo(π-C_3_Ph_3_)(CO)_2_], and its characterization by ^1^H NMR. The NMR spectrum showed a complex multiplet centered at δ = 7.2 ppm with peak intensity ratio 5:17 for the phenyl substituents of the cyclopropenium ring (Hayter, 1968). Since Hayter's report, other isolated cyclopropenylium-metal complexes were reported; Komatsu et al. summarized these in an extensive review published in 2003 (Komatsu and Kitagawa, 2003). To the best of our knowledge, no new π-complexes of this type have since been reported.

**Figure 7:**
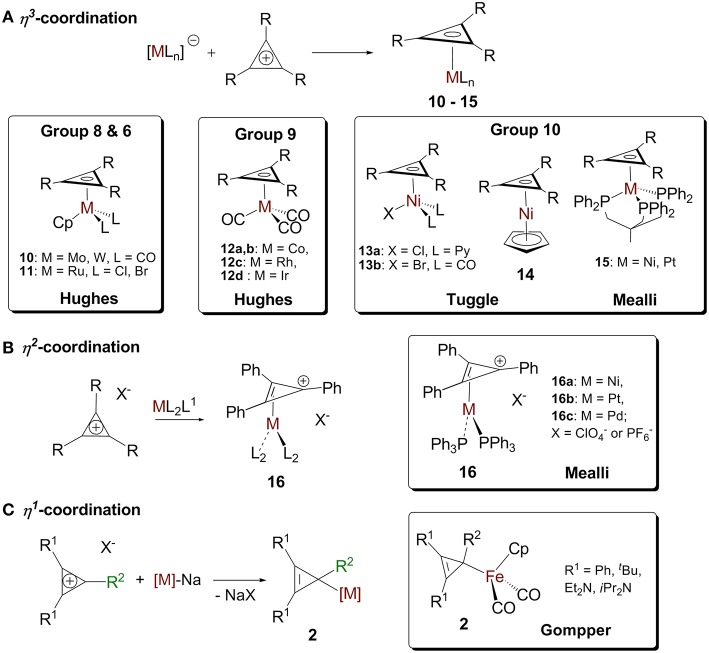
**(A)** η^3^-cyclopropenium complex. **(B)** d^10^ (η^2^-cyclopropenium) ML_2_ complex and the “Ring-whizzing” phenomenon. **(C)** η^1^-cyclopropenium complex.

The reactions of cyclopropenylium cations with low valent metal centers can lead to (η^3^-cyclopropenyl)- (Chiang et al., 1979; Hughes et al., 1986, 1993; Lichtenberger et al., 1993; Ghilardi et al., 1995), (η^2^-cyclopropenyl)- (Mealli et al., 1982), and (η^1^-cyclopropenyl) (Gompper and Bartmann, 1985) metal complexes. The hapticity of the product depends on the ring's substituents, the metal, and the other ligands on the metal (Figure 7).

**Figure 8:**
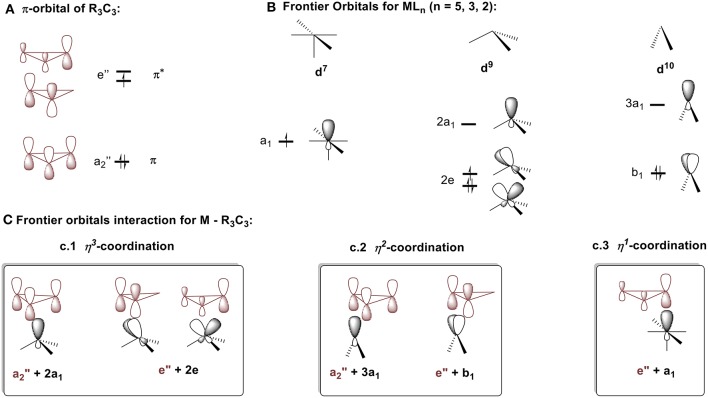
Descritpion of bonding mode between cyclopropenium and metal fragments. **(A)** π-orbital of R_3_C_3._ **(B)** Frontier orbitals ML_n_ (*n* = 5, 3, 2). **(C)** Frontier orbitals interaction for M – R_3_C_3_: (c.1) η^3^-coordination, (c.2) η^2^-coordination, (c.3) η^1^-coordination.

To better understand which coordination mode will be favored in each of these complex types, the molecular diagrams of C_3_R_3_ and the frontier orbitals for ML_n_ fragment (*n* = 2–5) (Jorgensen and Salem, 1973) are shown in Figures 8A,B respectively. The cyclopropenyl ring can either act as a: (1) σ-donor with its filled a_2_″ orbital; (2) π-acceptor with its empty e″ orbital, if bound in an η^2^ or η^3^ coordination mode; or (3) σ-acceptor with one of the empty e″ orbitals if bound in an η^1^ fashion. The coordination mode and the ring-metal interaction are determined by the d electron count of the metal and by the ligand environment of the metal fragment as established by a molecular orbital approach developed by Hoffmann et al. (Jemmis and Hoffmann, 1980). In short, the ML_n_ group will adopt the position that maximizes stabilizing bonding interactions. For the following discussion, the molecules will be arbitrarily split into neutral fragments, C_3_R_3_ and ML_n_.

##### η^3^ coordination

Cyclopropenium-metal complexes are prepared from the reaction between a cyclopropenium cation and a metal precursor, often a salt leading to the formation of a neutral species (Figure 7A; Donaldson and Hughes, 1982). We will model η^3^ coordination in cyclopropenium complexes with an ML_3_ d^9^ fragment, Co(CO)_3_. When the ML_3_ fragment is bound to the ring in this way (Figure 8c.1), the a_2_″ and e″ orbitals of the cyclopropenium moiety are symmetrical to the high-lying empty 2a_1_ orbital and the partially filled 2e orbital. In this case, coordination will optimize the number of metal-ring interactions, resulting in one σ- and two π-orbital interactions. If the [Co(CO)_3_]^−^ fragment moves off of the center of the ring and coordinates to it differently, part of the π-backdonation between the e orbitals will be lost. Evidence of the η^3^ bonding model for (C_3_R_3_)Co(CO)_3_ was confirmed experimentally by Lichtenberger et al. by photoelectron spectroscopy (Lichtenberger et al., 1993). This bonding model is applicable to complexes with the general formulas (C_3_R_3_)ML_3_ (where M = Co, Rh, Ir) (Chiang et al., 1979) and (C_3_R_3_)ML_2_X (where M = Ni, Pt, Pd; X = anionic ligand; L = neutral ligand) (Mealli et al., 1981, 1983; Miki et al., 1988; Kuchenbeiser et al., 2008). Since d^9^ ML_3_ and d^5^ ML_5_ complexes are isolobal, this η^3^ bonding is also appropriate for complexes with the general formula (C_3_R_3_)ML_3_X_2_ (where M = Ru) (Ditchfield et al., 1993; Morton and Selegue, 1999) and (C_3_R_3_)ML_4_X (where M = Mo or W) (Hayter, 1968; Drew et al., 1981; Hughes et al., 1985).

In general, the bonding mode in cyclopropenium-transition metal complexes depends on the metal involved. Strong π-backdonation from the metal to the carbocation increases the Z-type character of the ligand. It follows, then, that the distance between the metal and the C_3_ ring decreases while the Δ*G*^‡^ of rotation around their bond increases. If the metal in one of these complexes is coordinated to CO ligands, the bond between the metal and CO ligand increases in length, which results in a distinct shift in the υ_(CO)_ relative to that of the free CO-coordinated metal complex.

Infrared spectroscopy is the most powerful tool for monitoring reactions of metal carbonyls and for assignment of their structures, since the υ_(CO)_ absorptions are easily altered by changes to the molecular structure and charge of a carbonyl complex. It is unsurprising, then, that ion pairing between the cyclopropenium cation and a metal carbonyl anion results in significant changes in the υ_(CO)_ region, due to strong perturbation of the geometry of the anion. This characteristic is the result of π-backbonding, since CO is a π-acceptor ligand. When the υ_(CO)_ decreases as a result of decreasing bond strength, the strength of the M-C bond increases. As the M-C_3_ distance decreases, the M-CO distance increases; therefore, a longer M-C_3_ distance corresponds to a shorter M-CO distance and a decrease in υ_(CO)_ relative to free CO (2143 cm^−1^).

The υ_(CO)_ of a complex is affected by the nature of the substituents on the C_3_ ring (Table 1). When the ring substituents are changed from phenyls to t-butyls in the otherwise identical cobalt carbonyl complexes (**12a** and **12b**, respectively), the υ_(CO)_ decreases and the metal-centroid bond distance increase significantly as a result of the increase in electron donation from the substituent, consistent with an increase in pK_R_^+^ values (Table 1). It is worth mentioning that no metal complexes are reported with tris(amino)cyclopropenylium cations, suggesting that these are inadequately π-accepting (Table 1, pK_R_^+^ > 10) (Ciabattoni and Nathan, 1969; Moss et al., 1986; Bandar and Lambert, 2013; Jiang et al., 2015). The nature of the metal also affects the electronic configuration of the complex (Table 1). As expected, larger metals (Co to Ir in complexes **12b−12d**) provide better orbital overlap. More π-donation between the metal and the ring is observed from Co to Ir, which is consistent with the increase in υ_(CO)_. Finally, the extent of the back donation from the metal to the ring is dependent on the π-accepting ability of the other ligand bound to the metal. The nickel complexes in the table below (13a, 14, and 15) illustrate this: the trisphosphine (**15**) is a stronger π-accepting ligand than either Cp or two pyridines and a chloride (**14** and **13a**, respectively), and an increase in the M-C_centroid_ is observed. This suggests that the backdonation into the C_3_ ring is most significant in **13a**.

**Table 1:** Compiled M-C_3_ ring distance data (Churchill et al., 1984) and IR frequencies for CO in selected π-complexes.

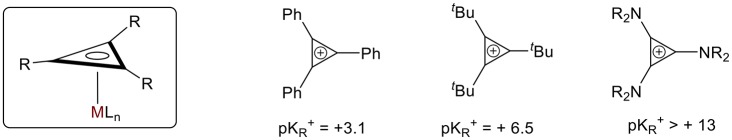			
**Complex**	**M-C_3_ centroid distance (Å)**	***υ*_(CO)_ cm^−1^**	**Metal υ_(CO)_ cm^−1^ (Ellis, 2003)**
**12a**: Co(C_3_Ph_3_)(CO)_3_	2.01 (Chiang et al., 1979)	2,080, 2,040 (Hughes et al., 1993)	[Co(CO)_4_]^−^ = 1,888
**12b:** Co(C_3_*^*t*^*Bu_3_)(CO)_3_	–	2,046, 1,976 (Hughes et al., 1993)	
**12c**: Rh(C_3_*^*t*^*Bu_3_)(CO)_3_	–	2,055, 1,991 (Hughes et al., 1993)	[Rh(CO)_4_]^−^ = 1,895
**12d:** Ir(C_3_*^*t*^*Bu_3_)(CO)_3_	2.02 (Hughes et al., 1993)	2,053, 1,985 (Shen et al., 1993)	[Ir(CO)_4_]^−^ = 1,895
**13a**: Ni(C_3_Ph_3_)Cl(py)_2_	1.94 (Tuggle and Weaver, 1971a)		
**14**: Ni(C_3_Ph_3_)(*η^5^*-C_5_H_5_)	1.96 (Tuggle and Weaver, 1971b)	–	–
**15:** Ni(C_3_Ph_3_)(CH_3_(CH_2_PPh_2_)_3_)	2.03	–	–

Tuggle and Weaver determined an important factor of the electronic transitions in their [(π-Ph_3_C_3_)NiCl(py)_2_]**·**py compound **10c** by comparing the UV-Vis spectra of their metal complex to that of the free ligand. Since the spectra showed no appreciable differences in their π → π^*^ transitions, they concluded the principle bonding interaction in the metal complex is not involved in the π → π^*^ transition (Tuggle and Weaver, 1971a). Later, they studied an analogous mixed nickel sandwich **11**, [(π-Ph_3_C_3_)Ni(π-C_5_H_5_)_2_], and considered the metal's interactions with each ring separately. They presented two possibilities for the cyclopropenyl moiety's interactions were presented: 1) overlap of a hybridized metal a_1_ orbital (with 3d_z_^2^, 4s, and 4p_z_ contributions) with the a_1_ combination of the C_3_ pπ orbitals; and (2) back-donation from the metal e orbitals to the e antibonding combination of the ring pπ orbitals. Importantly, they concluded that the back-donation was likely directed toward the formally positively charged C_3_ ring and not toward the cyclopentadiene (Tuggle and Weaver, 1971b). This finding is consistent with our conclusions above regarding M-C_3_ distance and υ_(CO)_.

Prior studies by Hughes et al. measured the free energy of activation (Δ*G*^‡^) for cyclopropenium ring rotation in Mo, Ru, Co, Rh, Ir derivatives (Hughes et al., 1993), which provided quantitative correlations between electronic and steric effects of ancillary ligands. Comparison of these experimental Δ*G*^‡^ values showed a significant increase in the rotational barrier of C_3_ rotation about the metal-C_3_ axis with descending group, which agreed with their prior findings in η^3^ complexes (Co < Rh < Ir) (Hughes et al., 1993) and with general observations made for rotational barriers of olefin and 1,3-diene complexes of transition metals (Mann, 1982).

##### η^2^ coordination

We will model η^2^ coordination with a d^10^ ML_2_ fragment, Ni(PPh_3_)_2_. In this case, only two frontier orbitals are suitable to interact with the ring (Figure 8c.2). According to Hoffmann and Mealli's calculations, the low energy levels consist of four closely spaced levels, b_2_+1a_1_+a_2_+2a_1_, which are identifiable with the e_g_ + b_2g_ + a_1g_ set of typical square planar ML_4_ systems (not shown in Figure 8B) (Jemmis and Hoffmann, 1980; Mealli et al., 1982). Higher in energy is b_1_, which is hybridized out away from the L groups and toward the cyclopropenium ring. Even higher in energy is 3a_1_, which is cylindrically symmetrical and is also hybridized away from L groups. The b_1_ orbital is the HOMO of a d^10^ ML_2_ fragment and the 3a_1_ orbital is the LUMO. The high lying empty 3a_1_ orbital can interact with the filled a_2_″ orbital of the ring and the filled b_1_ metal orbital can undergoes back donation with one of orbital of e″ set of the C_3_H_3_^+^ (Figure 8A). It is worth mentioning that π interaction between the low lying b_2_ metal orbital and the other component of the e″ orbital is present but much weaker. The loss of this π interaction is compensated by the metal fragment sliding in an η^2^ coordination mode to optimize the π interaction that involves the frontier b_1_ orbital of the metal. This bonding type will described for complexes of the type [(C_3_R_3_)ML_2_]^+^ (M = Ni, Pt, Pd) and [(C_3_R_3_)ML_4_]^+^ with a d^8^ metal due to the isolobal relationship of d^8^ ML_4_ and d^10^ ML_2_ complexes.

The clearest indication of η^2^ bonding is unequal distances between the metal atom and any of the three carbons in the ring. McClure and Weaver's platinum complex in 1973 was the first report of this unsymmetrical bonding (McClure and Weaver, 1973). In their complex, **16b** [Pt(C_3_Ph_3_)(PPh_3_)_2_][PF_6_] (McClure and Weaver, 1973), the Pt atom is 2.09 away from 2 of the cyclopropenium carbons, while it is 2.48 away from the third carbon. McClure concluded that his complexes are more closely related to the η^2^-cyclopropene resonance form and less like the η^3^ complexes Weaver synthesized earlier and that the coordination geometry and bond lengthening could be described with the bonding mode of olefins to zerovalent transition metals (McClure and Weaver, 1973).

In 1982, Mealli et al. published an important report on a phenomenon in which an ML_*n*_ unit migrates inside the periphery of a cyclic polyene. They called this unique fluxionality “ring-whizzing” (Mealli et al., 1982). Mealli compared three complexes **16** [(Ph_3_C_3_)M(PPh_3_)_2_]X (where M = Ni (**16a**), Pt (**16b**)or Pd (**16c**) and X = ClO_4_^-^ or PF_6_^-^) by ^13^C NMR and by computational studies informed by their X-ray structure data. They determined that the (Ph_3_P)_2_M unit progressively moved over the face of the cyclopropenium cation. This movement was used to chart the reaction path from one η^2^ geometry, with the (Ph_3_P)_2_M unit positioned below one C-C bond, to an equivalent η^2^ geometry. They concluded that a smaller distance between the metal and one of the carbons in the ring resulted in increased tilting and twisting of the phenyl group directly connected to it. These geometric changes caused longer exocyclic C-C distances because of the decreased conjugation between the phenyl groups and the cyclopropenium ring (Gompper and Bartmann, 1985).

##### η^1^ coordination

We will model the η^1^ coordination mode with a d^7^ ML_5_ fragment, [Fe(CO)_2_Cp]^−^ (Gompper and Bartmann, 1978). The frontier orbital of this fragment has two electrons in the a_1_ hybrid metal orbital that will interact with one component of the e″ cyclopropenium set. The low-lying filled e set of the metal will only have a small interaction with the e″ of the ring. The metal-ring interaction that contains only one σ molecular orbital will be strengthened if the fragment slides into an η^1^ mode (Figure 8c.3). This type of interaction is consistent with the model C described in Figure 4B. While synthesizing the first σ-complexes in 1978 (vide supra), Gompper and Bartmann synthesized a neutral intermediate (η^1^-cyclopropenyl)iron (C_3_R_3_)Fe(CO)_2_Cp complex **2** (Figure 7C; Gompper and Bartmann, 1978). This coordination mode is rare and is mostly reported as intermediate compounds in the reaction path to the formation of cyclopropylenium σ-complexes and will not be further discussed.

To conclude, (1) d^7^ ML_5_ complexes interact with C_3_R_3_^+^ with a single σ-type orbital, resulting in η^1^ coordination for [(C_3_R_3_)ML_5_] complexes, (2) d^10^ ML_2_ complexes have one σ and one π orbital interaction with C_3_R_3_^+^, supporting a η^2^ bonding mode in complexes with the formula [(C_3_R_3_)ML_2_]^+^, and (3) d^9^ ML_3_, and isolobal fragments (vide supra) have one σ and two π orbital interactions with the C_3_R_3_^+^, resulting in an η^3^ mode of coordination in complexes with the formula [(C_3_R_3_)ML_3_].

#### Arylenium Cations

**Figure 9:**
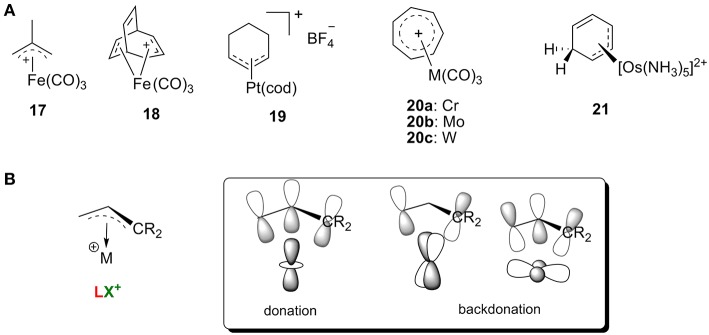
**(A)** Selective transition metal-stabilized carbocations with conjugated ϕ-system, and **(B)** the correlating orbital model.

Carbocations with conjugated π systems are one of the most common types of carbocation encountered in organic reactions, and iron was one of the earliest metals used to stabilize these carbocations (Olah et al., 2009). In general, the π orbitals of the carbocation can accept the backdonation of filled d orbitals on the metal atom, so the carbocations act as LX′ ligands. Figure 9B shows several representative carbocations with π-allylic systems complexed to an iron (**17** and **18**) in addition to π-allylic systems complexed to platinum (**19)**, chromium, molybdenum, or tungsten (**20)** and **an** arenium cation stabilized by Os complexation **21** (Green et al., 1977; Mayr et al., 1993; Winemiller et al., 1997). All of these complexes are consistent with the orbital models in Figure 9A.

In addition, these metal-stabilized arylenium cations can be easily characterized by their ^13^C NMR spectra. For example, complex **19** is featured with a typical resonance around 112 ppm for the central carbon of the η^3^-C_3_ system, along with two terminal carbon atoms of the allyl group around 82 ppm (Green et al., 1977)^.^ The osmium π-complex **21** was also characterized with three ^13^C resonances in the range of 75–85 ppm, indicating that the metal binds to the arenium system in an η^3^ fashion (Winemiller et al., 1997). Interestingly, studies showed that order of average reactivity of **20** toward nucleophiles was **20a**>**20b**>**20c** (Mayr et al., 1993), which can be rationalized with the orbital model in Figure 9A. Increasing the atomic radius leads to stronger backdonation of filled d orbitals on the metal atom (W>Mo>Cr) resulting in a metal π complex that is more stable and less reactive.

### Carbocations α to Cyclic π-Systems

**Figure 10:**
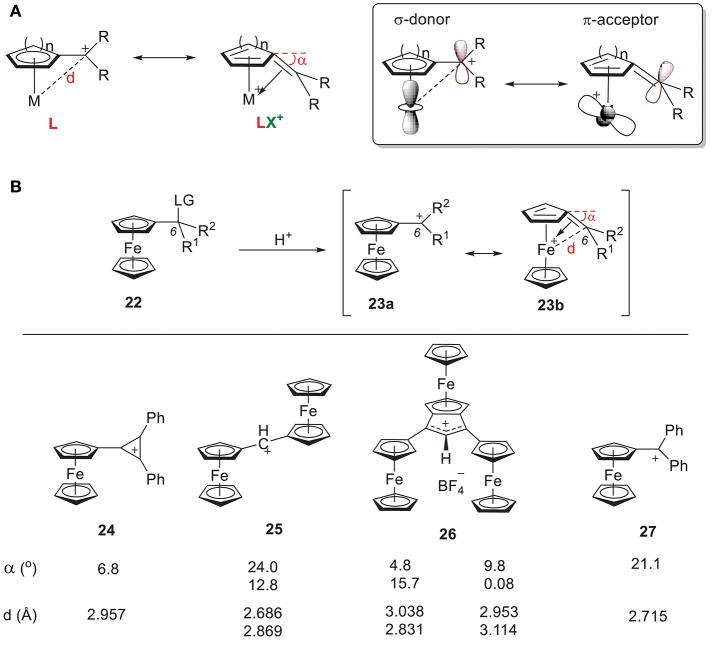
**(A)** Two resonance forms of α-metallocenylmethylium cation and their orbitals. **(B)** Selected α-metallocenylmethylium cations and their general approach.

Another extensively studied carbocation-metal complex is α-metallocenylmethylium cation (Hill and Richards, 1961; Davis et al., 1971; Gleiter et al., 2007; Bleiholder et al., 2009; Minić et al., 2015, 2017; Espinosa Ferao and García, 2017; Muratov et al., 2017; Preethalayam et al., 2017; Fomin et al., 2018). Two different resonance structures have been proposed: (1) the cation acts as an L-type ligand by donating its filled p orbital electron density to the metal center, and (2) the cation acts as an LX^+^ ligand by donating its filled p orbital electron density to the metal center through carbenium backdonation (Figure 10A). Consequently, some bending of the *sp*^2^-hybridized carbocationic center toward the metal atom has always been observed, which indicates the formation of double bond.

#### Carbenium α to Cyclopentadienyl

α-ferrocenyl carbenium ions were first observed by Richards and Hill (1959) through solvolysis of the corresponding ferrocenyl carbinyl acetate (Richards and Hill, 1959). Since then, a wide range of α-ferrocenyl carbenium ions have been synthesized and characterized under acidic conditions through their corresponding precusors (Figure 10B). Figure 10B shows some examples of reported α-ferrocenyl carbenium ions, such as tetrafluoroborates of ferrocenyl diphenyl cyclopropenium ion **24** (Sime and Sime, 1974), α,α'-diferrocenyl methylium ion **25** (Cais et al., 1978), ferrocene-annelated allylium ion **26** (Lukasser et al., 1995), and ferrocenyl diphenyl methylium ion **27** (Behrens, 1979), along with their geometric parameters as derived from X-ray diffraction studies. There is consistent bending of the C6 atom of the fulvene ring toward the iron atom in all of these complexes, but the bending angle (α) and Fe-C6 distance (d) vary considerably.

In **24**, the positive charge of the carbenium center is delocalized into the cyclopropenylium ring, which results in a bending angle of 6.8° with an Fe-C6 bond length of 2.96 Å. In **25**-**27**, an increase of the angle (α) and a reduction of the Fe-C6 distance (d) is observed. These geometric changes can be rationalized by the orbital model in Figure 10A. The π acidity of the C6 center depends on the identity of R^1^ and R^2^. Greater electron density in the carbenium center results in less π acidity, which results in weaker π backdonation and a conformation like **23b** with a smaller bending angle. For example, the π systems in **24** and **26** increase the electron density of their C6 centers, yielding smaller bending angles.

**Figure 11:**
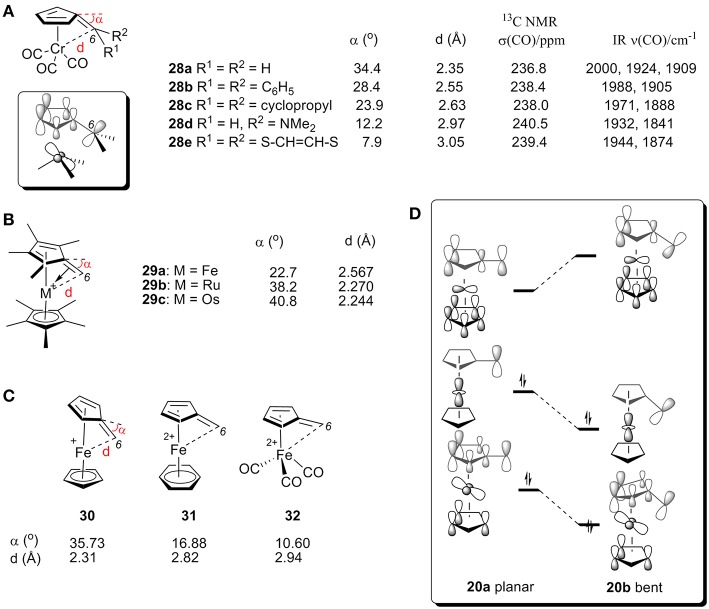
**(A)** X-ray, ^13^C NMR, and IR data for CO of complexes **28**. **(B)** Geometric parameters of complex **29** by X-ray analysis. **(C)** Geometric parameters of complexes **30-32** by DFT calculations. **(D)** Diagram between the frontier orbitals of a planar and bent α-ferrocenyl methylium ion.

[(η^6^-C_5_H_4_C(C_6_H_5_)_2_)]Cr(CO_3_) (**28b**) was the first η^6^-fulvene complex studied by X-ray analysis (Figure 11A). This data experimentally confirmed the predicted tendency toward strong bending of C6 (Andrianov et al., 1975). The neutral complex **28b** shows a bending angle of 28.4° with a Cr-C6 bond length of 2.55 Å, which can be explained by the HOMO obtained from extended Hückel calculations is shown in Figure 11D (Albright et al., 1978). The bending of C6 causes a bonding interaction between C6 and the Cr-centered e_s_ orbital (Albright et al., 1978). X-ray studies of various fulvene-Cr(CO)_3_ complexes with different substituent groups at C6 (**28a**-**e**, Figure 11A) showed the strongest bending in the unsubstituted fulvene ligand (**28a**) and only a small amount of tilt angle for **28e**, which contains a conjugated 6π-electron system at the C6 atom of the fulvene ligand (Behrens, 1979; Lubke et al., 1983). Fulvene-Cr(CO)_3_ complexes with different groups at C6 also showed an impact on the CO chemical shift of ^13^C NMR and stretching frequencies by IR (Lubke et al., 1983). The bending angle and C6-Cr distance changes are consistent with the above α-ferrocenylmethylium ions. In general, electron-donating groups increase the electron density of the C6 center, thereby increasing σ-donation and decreasing carbenium backdonation, resulting in smaller tilt angle (α), larger CO chemical shift (δ) and lower υ_(CO)_.

X-ray studies also showed that α-metallocenyl methylium cations show an increased metal-fulvene interaction with an increase in the metal's molecular mass. The bending angles for complexes **29a** (Kreindlin et al., 2000), **29b** (Kreindlin et al., 1987), and **29c** (Rybinskaya et al., 1989) are 22.7°, 38.2°, and 40.8°, respectively, (Figure 11B), which indicates a dramatic increase in metal-fulvene interactions. This is because a larger atomic radius leads to more overlap of filled metal d orbitals with the carbenium p orbital, resulting in strong carbenium backdonation and a large tilt angle (Figure 10A).

The effect of ligands has also been studied with DFT calculations (Figure 11D; Gleiter et al., 2007). We can see from MO diagrams that electron-rich ligands favor interactions with metal and carbenium centers [Figure 11C, Cp (**30**) > benzene (**31**) > CO (**32**)]. According to the orbital model, the electron-rich ligand can increase the electron density on the metal atom, resulting in carbenium backdonation and a large tilt angle.

Figure 11D shows the correlated frontier orbitals of a planar (left) and a bent structure (right) of α-ferrocenyl methylium ion (Gleiter et al., 2007; Bleiholder et al., 2009). When the bending angle increases, the LUMO is destabilized and the HOMO is stabilized, which can be rationalized by the increased bonding interaction between the C6 p orbital and the metal d-orbital of the HOMO and an increased antibonding interaction between the C6 and metal orbitals of the LUMO. As a result, electron density is transferred into an antibonding orbital between C1 and C6, resulting in a larger bending angle (α). Additionally, increasing the electron density of the metal center (e.g., heavier metal or electron-rich ligand) or decreasing the electron density of the carbenium center (e.g., electron-withdrawing group) will favor these interactions, leading to larger bending angle α.

#### Carbenium α to Aryl

**Figure 12:**
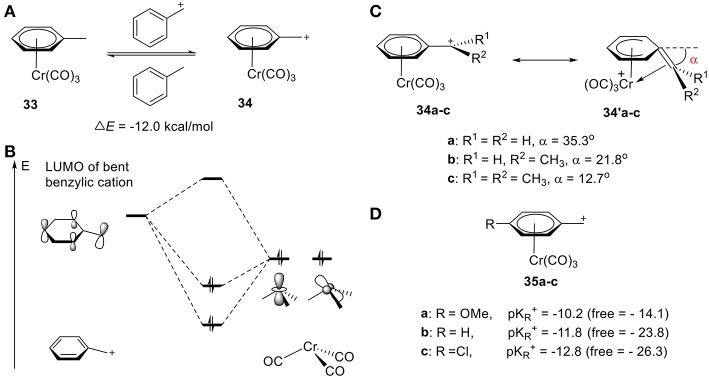
**(A)** Chromium-stabilized carbocations. **(B)** Selected stabilizing orbital interactions between Cr(CO)_3_ and benzylic cation. **(C)** Two resonance forms of Chromium-stabilized benzylic cations according to DFT calculation. **(D)** pK_R_^+^ values of different Cr-stabilized benzylic cations.

Cr is well-established in its ability to stabilize carbocations, including benzylic, phenonium, and benzonorbornenyl cations (Tantillo et al., 2000; Merlic et al., 2001b; Konietzny et al., 2010; Davis et al., 2013). In 1999, the groups of Houk (Merlic et al., 1999) and Koch (Pfletschinger et al., 1999) pioneered the field through theoretical computations determining the stabilization of benzylic cations by chromium tricarbonyl. As shown in Figure 12A, the homodesmotic equation gives a Δ*E* of −12.0 kcal/mol, suggesting effective stabilization of the benzylic cation **34** by Cr(CO)_3_ (Figure 12A). This stability is attributed to the other resonance form **34′**, in which the benzylic carbon bends down to coordinate to the Cr atom with an angle of 35.3°, 21.8°, and 12.7°, according to DFT calculations, for methyl, ethyl, and isopropyl cation, respectively, (Figure 12C; Merlic et al., 2001a). This is consistent with the substituent effects observed in Figure 11A. Electron-donating groups result in a smaller tilt angle (α), though steric repulsions are also likely contributive. The stability can also be rationalized in terms of orbital interactions between the hybrid fragment orbitals (Albright, 1982) of Cr(CO)_3_ and the π molecular orbitals of benzylic cation (Figure 12B; Merlic et al., 2001a). In the case of the cation, the low-lying LUMO interacts strongly with the symmetric occupied hybrid metal orbitals. The overlap between the Cr and benzylic cation orbitals (especially the d_z_^2^-like metal orbital) is increased in two ways: (1) the distortion of the benzylic cation from planarity and, (2) shifting the chromium away from the center of the ring. Electron-donating groups can increase the electron density of C_α_ center and decrease carbenium backdonation, leading to a smaller tilt angle (α). These computed results are supported by the experimental pK_R_^+^ values of Cr-stabilized benzyl complexes **35a-c** (Figure 12D). The pK_R_^+^ increased sharply as compared to the corresponding free carbocations, indicating the stabilization of the carbocation by the Cr atom (Figure 12D). Additionally, more electron-deficient benzyl moieties (electron withdrawing groups) gain an even greater stabilization effect through increased π-backdonation (Trahanovsky and Wells, 1969; Cheng et al., 1993).

### Carbocations α to Acyclic π-Systems (alkynyl)

**Figure 13:**
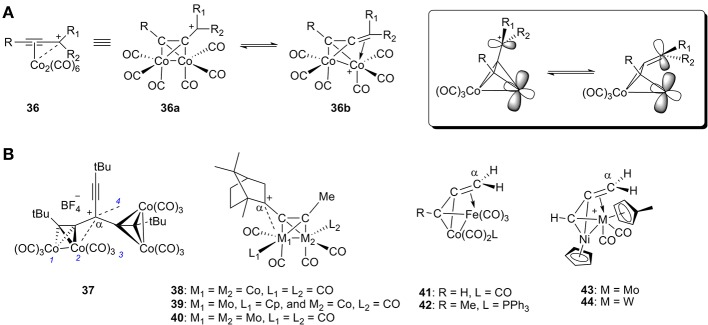
**(A)** Two resonance forms of α-metallocenylmethylium cation and their orbitals. **(B)** Selected representative cationic bimetallic propargyl complexes.

Cobalt, especially cationic dicobalt propargyl complexes, have played a significant role in organic synthesis since their discovery (Nicholas and Pettit, 1971; Nicholas, 1987; McGlinchey et al., 1995; El Amouri and Gruselle, 1996; Amouri et al., 2000). In general, there are two resonance forms for this kind of propargyl cation complex **36**: (1) the cation acts as an L-type ligand by donating its filled π orbital electron density to the metal center, and (2) the cation acts as a LX^+^ ligand by donating its filled π orbital electron density to the metal center with carbenium backdonation (Figure 13A). Both of these resonance forms provide stability to the carbocation.

A wide range of cationic dicobalt propargyl complexes or similar heterobimetallic complexes have been synthesized and characterized (**37**-**44**, Figure 13B; Gruselle et al., 1993; Osella et al., 1993; Melikyan et al., 1998; Chetcuti and McDonald, 2002). The propargyl cation always preferentially coordinates to one of the metal atoms in each cluster due to accumulation of positive charge. In studies of these heterobimetallic complexes, the propargyl cation prefers to coordinate to Mo and Fe instead of Co (**39**, **41**, and **42**) (Gruselle et al., 1993; Osella et al., 1993) as well as Mo and W instead of Ni (**43** and **44**) (Chetcuti and McDonald, 2002).

#### X-Ray Crystallography

Table 2 gives a summary of the M-C_α_ distance of the bimetallic complexes discussed above. In **37**, the distances between the carbocationic center and the cobalt atom are 3.07, 2.81, 3.27, and 2.89 Å, respectively, for Co1, Co2, Co3, and Co4. The 2-bornyl cation leans toward the Mo atom and the Mo-C_α_ distance is 2.74 Å for **39** and 2.91 Å for **40**. The preferential stabilization of the 2-bornyl cation by the molybdenum has also been rationalized with molecular orbital calculations at the extended Hückel level (Gruselle et al., 1993). Cations **38**-**40** do not undergo Wagner-Meerwein rearrangement as a result of their stabilization, which otherwise occurs readily for uncomplexed 2-alkynylbornyl cations. For **42**, the Fe-C_α_ distance is 2.195 Å. The preferential coordination of C_α_ with Fe has been explained by the model cluster **41** by means of extended Hückel molecular orbital calculations (Osella et al., 1993).

**Table 2:** X-ray, IR, and ^13^C NMR data for the complexes.

**Complex**	**M-C_α_ distances (Å)**	**IR ν_CO_ (cm^−1^) in DCM**	**IR ν_CO_ (cm^−1^) in Hexane**	**^13^C NMR δ(CO), ppm**
**37**	Co1: 3.07 Co2: 2.81 Co3: 3.27 Co4: 2.89	–	–	194.4, 192.2
**38**	–	–	–	194.4, 192.2
**39**	2.74	–	–	–
**40**	2.91	–	–	227.1, 226.5, 223.0, 220.0
**41**	–	–	2,084 m, 2,041 vs. 2022 s, 2,014 m, 1,993 m, 1,981 m	210.3, Fe(CO)_3_; 203.0, Co(CO)_3_
**42**	2.20	–	2,052 vs. 2,003 s, 1,989 vs. 1,968 m	211.2, Fe(CO)_3_; 207.3, 205.1, Co(CO)_2_
**43**	–	2,049 s, 2,019 s, 1,966 m	–	–
**44**	–	2,047 m, 2,013 s, 1,989 w, 1,966 vw	–	–

#### IR and ^13^C NMR Spectroscopy

Table 2 also gives a summary of the IR and ^13^C NMR data of the above bimetallic complexes. Generally, the IR ν_CO_ stretching frequencies of these cations are shifted to higher values (over 2000 cm^−1^) compared to their corresponding neutral precusors (Osella et al., 1993; Chetcuti and McDonald, 2002). In dicobalt cation **27**, the ^13^C NMR CO signals appear at approximately 194 ppm. Analogously, stabilization of a propargyl cation by a molybdenum center results in a shielding of the molybdenum carbonyl signals from approximately 230–220 ppm in **40** (Gruselle et al., 1993). For **41** and **42**, stabilization of a propargyl cation by an iron center shifts the carbonyl resonances to approximately 210 ppm, while the cobalt carbonyl resonances are around 203–208 ppm (Osella et al., 1993).

This stabilization can be explained in terms of orbital interactions between the metal's d orbital and the π molecular orbitals of the propargyl cation in Figure 13A (Gruselle et al., 1993; McGlinchey et al., 1995). The overlap between the orbitals of the metal center and of the propargyl cation (especially the d_z_^2^-like metal orbital) is increased by the shorter M-C_α_ distance, resulting from the propargyl cation bending toward the metal. The preferential coordination with a heavier metal within the heterobimetallic cations **39, 40**, and **43** is consistent with the reactivities of complex **20** and the conclusion in Figure 9B. However, in complexes **41-43**, the carbocation is bound to the lighter element, Fe. This can be explained by considering the isolobal relationship between Co(CO)_3_^+^ and Fe(CO)_3_ (McGlinchey et al., 1995). The neutral Fe can provide more effective overlap of filled metal d orbitals with the carbenium's p orbital than Co^+^, which makes the interaction with Fe more attractive. The coordination of the metal with the cation also induced higher CO stretching frequencies in IR and larger chemical shifts for CO in ^13^C NMR.

## Conclusion

We proposed two major bonding modes for the orbital interactions between carbeniums and metal centers in σ- and π-complexes. Most of the reported transition metal-carbocation complexes can fall into one of these two categories. In general, heavier metal atoms have larger radii, which can lead to stronger backdonation of filled d orbitals on the metal atom and greater stabilization of carbocations. In addition, electron-donating groups on the carbocations can increase the electron density of carbon center, thus increasing σ-donation while decreasing carbenium backdonation. This results in weaker transition metal-carbocation interactions. On the other hand, an electron-rich ligand can increase the electron density on the metal atom, resulting in carbenium backdonation and greater stabilization. The stabilization of carbocations by the transition metal has been unambiguously demonstrated with higher pK_R_^+^ values in comparison to the corresponding free carbocations.

Transition metal-stabilized carbocations have been observed and characterized throughout the last century, but there is no comprehensive summary of the bonding modes of these transition metal-carbocation complexes. To our surprise, most of this research was conducted and reported before 2000 and little attention has been given to the field during the last decades, even though much remains unknown about their properties, reactivities, and carbocation interactions with other transition metals (e.g., Pd, Rh, Ir, Ni). Because of their considerable synthetic value, it is of great importance to bring these metal-carbocation interactions back to the interest of the scientific community.

## Author Contributions

All authors listed have made a substantial, direct and intellectual contribution to the work, and approved it for publication.

### Conflict of Interest Statement

The authors declare that the research was conducted in the absence of any commercial or financial relationships that could be construed as a potential conflict of interest.
